# Family Functioning and Its Predictors among Disaster Bereaved Individuals in China: Eighteen Months after the Wenchuan Earthquake

**DOI:** 10.1371/journal.pone.0060738

**Published:** 2013-04-03

**Authors:** Xiaoyi Cao, Xiaolian Jiang, Xiaolin Li, Man-chun Jenny Hui Lo, Rong Li

**Affiliations:** 1 Department of Nursing, West China Hospital, West China School of Medicine, Sichuan University, Chengdu, Sichuan Province, People’s Republic of China; 2 School of Social Science, Hong Kong Polytechnic University, Hong Kong, People’s Republic of China; Max Planck Institute of Psychiatry, Germany

## Abstract

**Background:**

The 2008 Wenchuan earthquake in China resulted in great loss of life and property, and previous studies have focused on psychopathological symptoms in survivors after disasters. This study examined perceived family functioning and its predictors in disaster bereaved individuals eighteen months after the 2008 Wenchuan earthquake.

**Methodology/Findings:**

This was a cross-sectional study of a convenience sample of 264 bereaved individuals. The instruments used in the study included Family APGAR Index, Family Adaptability and Cohesion Evaluation Scale??, Emotional and Social Loneliness Scale, and a range of items eliciting demographic characteristics and disaster-related variables. The results indicated that the rates of moderate family dysfunction and severe family dysfunction in bereaved individuals were 37.1% and 12.9%, respectively. Less financial loss during the earthquake was a significant predictor for positive family function. Better self-rated health status after the earthquake was significantly related to positive family function, cohesion, and adaptability. Scores on family cohesion and adaptability in bereaved individuals from extended or nuclear families were significantly higher than those from single-parent families. The ability to give birth to another baby of bereaved parents was a significant predictor for positive family function and cohesion. Poorer family function, cohesion and adaptability were significantly related to greater loneliness.

**Conclusions/Significance:**

This study found a high prevalence of family dysfunction in bereaved individuals eighteen months after the 2008 Wenchuan earthquake. Strategies can be designed to facilitate post-disaster recovery, particularly for the bereaved at high risk for family dysfunction. The study provides useful information for post-disaster rebuilding and relief work.

## Introduction

Natural disasters can occur rapidly and unpredictably leading to great loss of life and property, increased resource consumption within the family, and family dysfunction [1], [2]. Family dysfunction can in turn result in a range of psychological and behavioral problems, such as alcohol and drug abuse, depression and post traumatic stress disorder (PTSD) [3], [4]. For example, Rowe et al. (2010) found that lower family cohesion was a significant risk factor for substance involvement and post traumatic stress in adolescent survivors [4]. Similarly, Wickrama and Wickrama (2008) found that intact family status and family support reduced depression [5]. The results of a qualitative study indicated that, the essential task of damaged families after disasters is to rebuild family life, reduce uncertainty related to disaster, and improve family functioning [6].

Family functioning is defined as the extent to which a family operates as a unit to cope with stressors [7]. The Circumplex model of marital and family systems holds that family functioning is composed of three dimensions: cohesion, adaptability, and communication. Family cohesion refers to the emotional bonding among family members. Family adaptability represents the ability of a family to change its rules, role relationships, and power structure in response to developmental changes or situational stressors. Communication is a facilitating factor to cohesion and adaptability [8]. A review of literature indicates that a great majority of previous studies on family functioning have focused on patients, caregivers, children, and adolescents. Known predictors for family functioning include demographic characteristics, such as gender, age, marital status, educational level, occupation, and economic status [9], [10]. Meanwhile, family variables such as family structure, family support and coping style are also identified as significant predictors for family functioning [11], [12]. In addition, many studies have explored the effects of family functioning on behavioral and psychological problems, such as eating disorder, suicide, depression, and loneliness [13]–[17]. Natural disasters can result in deaths of family members, damage to family properties and loss of family security [18]. Disaster exposure can generate more proximal secondary disaster risks, such as post-disaster family problems [5]. For instance, it was reported that 28.6% of disaster-affected families experienced poor family resilience after Cyclone Larry in Australia [2]. However, to our knowledge, few studies have examined perceived family functioning in disaster survivors.

Bereavement caused by disasters often results in long-term psychological disorders. For example, a study of bereaved survivors two years after the 2004 Tsunami found that the rates of major depression and PTSD were 25.0% and 34.4%, respectively [19]. Another study of bereaved survivors in the 2008 Sichuan earthquake found that the prevalence of depression was 65.6% and the loss of a child was a significant predictor for psychopathological symptoms [20]. ABC-X model of family stress maintains that whether families survive or fall into X (the crisis) when dealing with situational stressors depends on three factors: A (the event), B (the family’s crisis-coping resources), and C (the family’s evaluation of the event) [21]. Because bereavement caused by disasters is a mass stressor, bereaved families without sufficient crisis-coping resources, such as adaptive family functioning, are likely to experience detrimental long-term outcomes after disasters. Furthermore, positive family functioning may be a protecting factor for mental health. However, no study has examined perceived family functioning and its related factors in disaster bereaved individuals.

In addition, loneliness defined as a chronic distress without redeeming features includes emotional loneliness and social loneliness [22]. It has been shown that absence of frequent interactions with family members, neighbors or friends, and small social networks can cause loneliness [23]. Studies on loneliness have found that demographic characteristics and personality variables are significant predictors for loneliness [24], [25]. However, these studies have focused on older people and patients with AIDS. Moreover, bereavement as a stressful event can disrupt family life and family functioning, which may in turn result in loneliness. However, no study has investigated the relationship between family functioning and loneliness in disaster bereaved individuals.

On May 12, 2008, an 8.0-magnitude earthquake occurred in the northwest of Sichuan province, China. As one of the most severe natural disasters in the history of China, it is reported that 69,227 individuals died, 374,643 individuals were injured and 17,824 individuals were missing in the disaster-affected regions [26]. A great number of survivors lost their beloved family members in the catastrophic event, and bereavement may have led to severe damage to family structure and family dysfunction. However, no study has examined perceived family functioning in bereaved individuals after disasters. Therefore, the aims of this study were to 1) examine perceived family functioning in bereaved individuals eighteen months after the 2008 Wenchuan earthquake; 2) explore the effects of demographic characteristics and disaster-related variables on family functioning; 3) analyze the relationship between perceived family functioning and loneliness.

## Methods

### Samples

A cross-sectional research design was used in the study. A convenience sample of 274 bereaved individuals was recruited via door-to-door interviews (each individual was drawn from a different bereaved family). Participants lived in the three hardest-hit regions in the Wenchuan earthquake (Dujiangyan, Mianzu and Li county) and all of them resided in the rural areas. The inclusion criteria were as follows: 1) the loss of biological family members in this earthquake (e.g., spouse, children, grandchildren, parents, grandparents and siblings); 2) aged 18 and older; 3) agreed to participate in the study. Participants who had cognitive impairment were excluded. The Chinese Mini–Mental State Examination (MMSE) was used to screen for cognitive impairment. According to the results, three different cut-off points were used depending on the respondent's educational level with a score >17 (illiteracy), >20 (primary school), and >24 (junior high school or above) indicating no cognitive impairment [27]. As a result, 10 potential participants were excluded based on their MMSE scores.

### Measures

#### Family APGAR Index

The scale assesses the extent to which individuals are satisfied with their family functioning [7], [9]. It is composed of five items: adaptation, partnership, growth, affection, and resolution with a three-point scale ranging from 0 *(hardly ever)* to 2 *(almost always)*. The total scores range from 0 to 10 with higher scores indicating higher levels of satisfaction with family functioning. A score of 0–3 indicates severe family dysfunction, 4–7 moderate family dysfunction, and 8–10 positive family function [28]. Cronbach’s alpha coefficients reported across studies ranged from 0.80 to 0.85 [29], [30]. In the study, Cronbach’s alpha coefficient for this scale was 0.88.

#### Family Adaptability and Cohesion Evaluation Scale α (FACESα)

The scale includes 30 items and two dimensions: family cohesion (16 items) and family adaptability (14 items) [8], [31]. It is evaluated by a five-point scale ranging from 1 *(almost never)* to 5 *(almost always)*. The total scores on family cohesion and adaptability range from 15 to 80 and 15 to 70 respectively, with higher scores suggesting better family cohesion and adaptability. Four family types are determined by participants’ combined scores on cohesion and adaptability subscales, namely balanced family type (cohesion: 71–80; adaptability: 55–70), moderately balanced family type (cohesion: 60–70; adaptability: 46–54), mid-range family type (cohesion: 51–59; adaptability: 40–45), and extreme family type (cohesion: 15–50; adaptability: 15–39). Cronbach’s alpha coefficient for the overall scale is 0.90, and test-retest reliability is 0.84 [32]. In the present study, Cronbach’s alpha coefficient for this scale was 0.82.

#### Emotional and Social Loneliness Scale (ESLS)

The 10-item scale consists of two subscales: emotional loneliness and social loneliness [33], [34]. Each subscale includes five items. A five-point scale from 1 *(hardly ever)* to 5 *(often)* is used. The total score for each subscale ranges from 5 to 25 with higher scores indicating greater loneliness. Cronbach’s alpha coefficients for emotional loneliness and social loneliness are 0.78 and 0.76, respectively. Examination of the relationship between the ESLS and UCLA (University of California, Los Angeles Loneliness Scale) demonstrated its good concurrent validity [35]. In the present study, Cronbach’s alpha coefficients for emotional and social loneliness were 0.76 and 0.90, respectively.

Referring to recent studies on natural disasters [20], [36], a number of items with fixed responses were developed in this study to evaluate disaster-related experiences during the Wenchuan earthquake, including types of dead family members in the earthquake, financial loss during the earthquake, housing conditions, types of family structure, self-rated health status and fecundity status after the earthquake. For instance, the items related to types of dead family members were: did your biological family members die in the earthquake? If yes, which family members died in the earthquake? The choices were spouse, children, grandchildren, parents, grandparents, and siblings. Responses to these choices were dichotomous (yes/no). With regard to the post-earthquake fecundity status of bereaved parents, the following four items were used: did your children die in the earthquake? If yes, did you and your spouse want another baby after the earthquake? If yes, did you and your spouse have another baby after the earthquake? If no, were you or your spouse pregnant after the earthquake? Responses to the above four items were also dichotomous (yes/no).

In addition, we also collected demographic data such as gender, age, religious beliefs, educational level, and marital status.

### Procedure

Prior to this study, two research assistants each with a master’s degree in nursing science participated in two training sessions to ensure that the measures would be administered reliably. As disaster-related experiences would be explored in bereaved individuals during the investigation, two experienced psychologists were invited to train the two research assistants effective communication skills to minimize respondents’ emotional distress. Three weeks after training, a pilot study was conducted on 23 bereaved individuals in Mianzu under the supervision of the psychologists. It was found that the data collection method was feasible, the two research assistants were able to make effective communication with the bereaved, and all the respondents understood the questionnaires.

After contacting with the local governments and community service organizations, this survey was carried out from December 1, 2009 to January 31, 2010 in Mianzu, Dujiangyan and Li county, which were the hardest-hit areas in the Wenchuan earthquake. Respondents were required to complete the questionnaires independently according to their actual feelings. With regard to respondents who could not complete the questionnaires by themselves due to physical illnesses, the investigators recorded their responses. As for those respondents who were illiterate, the investigators read the questions and responses word-for-word and recorded their answers. The questionnaires were collected immediately after completion and checked for incomplete items.

### Data analyses

The statistical analysis package used in the study was SPSS 16.0. Means and standard deviations (SD) on family functioning were examined. Independent samples t-tests or one-way analyses of variance were used to test differences in family functioning scores between two or more subgroups. Three multivariate regression analyses were run to identify significant predictors for family functioning, with family function, cohesion, and adaptability as the dependent variable, respectively, and demographic characteristics and disaster-related variables as the independent variables for each analysis. Among these independent variables, types of dead family members in the earthquake (reference group: other family members: parents, grandparents, siblings and grandchildren), types of family structure after the earthquake (reference group: single-parent family), and post-earthquake fecundity status of bereaved parents (reference group: not being pregnant) were included in regression equation as dummy variables. The relationships between family function, cohesion, adaptability and loneliness were evaluated by Pearson correlation analyses. *P* value < 0.05 was considered statistically significant.

### Ethical statement

Prior to the investigation, ethical approval was obtained from the Human Subjects Ethics Sub-committee of Sichuan University. Informed consent was obtained from each participant. Participants were assured of anonymity, confidentiality and their rights to withdraw from the study at any time.

## Results

### Characteristics of the sample

Of the 264 respondents in the study, 45.1% were male and 54.9% were female. The age ranged from 16 to 98 with an average age of 45.87 years (SD  =  13.08). The majority of the respondents were married (90.5%). 131 respondents (49.6%) experienced severe financial loss in the earthquake and 115 respondents (43.6%) lived in temporary post-disaster houses. The rates of post-earthquake nuclear family and extended family were 32.6% and 61.0%, respectively. In the earthquake, 142 respondents (53.8%) only lost their children, 15 respondents (5.7%) only lost their spouses, 57 respondents (21.6%) only lost other family members, such as parents, siblings, grandparents, or grandchildren, and 50 respondents (18.9%) lost two or more than two types of biological family members (Table 1). Overall, 190 respondents lost their children in the earthquake and 91.2% of the respondents (177/190) wanted another baby.

**Table 1:** Characteristics of respondents and scores on family function, cohesion and adaptability among different subgroups (n  =  264).

Variables	No. (%)	Family function	Family cohesion	Family adaptability
		Mean (SD)	Mean (SD)	Mean (SD)
Demographic characteristics				
Gender				
Male	118 (45.1)	6.52 (2.68)	64.31 (8.17)	41.58 (6.06)
Female	146 (54.9)	6.51 (2.65)	64.14 (9.95)	42.05 (7.06)
*P* value (t statistics)		0.99 (0.01)	0.89 (0.14)	0.57 (0.57)
Age (years)				
18–44	164 (62.1)	6.84 (2.50)	65.71 (8.36)	42.34 (5.87)
45–59	48 (18.2)	6.52 (2.71)	62.71 (10.92)	40.81 (7.82)
≥60	50 (18.9)	5.44 (2.92)	60.68 (8.73)	40.86 (7.12)
*P* value (F statistics)		0.01 (5.43)	0.00 (6.86)	0.20 (1.64)
Religious beliefs				
No	178 (67.4)	6.81 (2.55)	65.21 (8.67)	42.66 (6.47)
Buddhism	86 (32.6)	5.90 (2.78)	62.15 (9.88)	40.16 (6.65)
*P* value (t statistics)		0.01 (2.57)	0.01 (2.57)	0.00 (2.91)
Educational level				
Illiteracy	59 (22.3)	5.41 (2.78)	60.88 (10.33)	40.24 (6.90)
Primary school	83 (31.4)	6.39 (2.47)	63.00 (8.82)	41.28 (7.02)
Junior high school	109 (41.3)	7.19 (2.60)	67.13 (7.94)	43.16 (5.72)
High school or above	13 (5.0)	6.69 (2.10)	64.02 (9.18)	41.77 (8.32)
*P* value (F statistics)		0.00 (6.22)	0.00 (7.34)	0.04 (2.85)
Marital status				
Married	239 (90.5)	6.68 (2.50)	65.14 (7.98)	42.13 (5.96)
Single (unmarried/divorced/windowed)	25 (9.5)	4.92 (3.53)	55.36 (14.22)	39.08 (10.92)
*P* value (t statistics)		0.00 (3.21)	0.00 (5.32)	0.03 (2.21)
Disaster-related variables				
Financial loss during the earthquake				
Slight	48 (18.2)	7.62 (2.27)	66.71 (7.61)	43.98 (5.16)
Moderate	85 (32.2)	6.96 (2.38)	65.41 (7.80)	42.12 (6.46)
Severe	131 (49.6)	5.82 (2.77)	62.53 (12.20)	40.89 (6.62)
*P* value (F statistics)		0.00 (10.63)	0.01 (4.85)	0.02 (4.03)
Types of dead family members in the earthquake				
Two or more than two types of family members	50 (18.9)	6.32 (2.85)	63.00 (10.09)	40.14 (7.85)
Spouse	15 (5.7)	6.27 (3.37)	57.27 (15.83)	40.47 (10.59)
Children	142 (53.8)	6.90 (2.48)	65.89 (7.84)	42.76 (5.42)
Other family members (parents, siblings, grandparents and grandchildren)	57 (21.6)	5.79 (2.60)	62.93 (8.12)	41.42 (6.66)
*P* value (F statistics)		0.05 (2.60)	0.00 (5.37)	0.07 (2.34)
Post-earthquake housing conditions				
Permanent post-disaster houses	149 (56.4)	6.89 (2.24)	66.02 (7.14)	42.63 (5.34)
Temporary post-disaster houses	115 (43.6)	6.03 (3.06)	61.88 (10.88)	40.83 (7.89)
*P* value (t statistics)		0.01 (2.66)	0.00 (3.72)	0.03 (2.21)
Post-earthquake self-rated health status				
Good	116 (43.9)	7.28 (2.44)	67.02 (7.15)	43.40 (5.76)
Moderate	120 (45.5)	6.18 (2.66)	62.87 (9.31)	40.96 (6.78)
Poor	28 (10.6)	4.82 (2.47)	58.39 (11.97)	39.21 (7.87)
*P* value (F statistics)		0.00 (12.42)	0.00 (13.51)	0.00 (6.77)
Post-earthquake types of family structure				
Nuclear family	83 (32.6)	6.83 (2.58)	66.20 (9.05)	43.14 (6.50)
Extended family	161 (61.0)	6.52 (2.59)	64.73 (7.94)	41.95 (5.95)
Single-parent family	20 (6.4)	5.15 (3.15)	51.85 (10.06)	35.60 (8.83)
*P* value (F statistics)		0.04 (3.28)	0.00 (23.89)	0.00 (11.35)
Post-earthquake fecundity status				
Not pregnant	80 (45.2)	6.69 (2.40)	64.61 (7.81)	41.70 (5.86)
Pregnant	74 (41.8)	6.80 (2.32)	65.84 (7.10)	42.80 (4.81)
Already having another baby	23 (13.0)	8.04 (2.10)	70.09 (7.00)	44.48 (6.16)
*P* value (F statistics)		0.04 (3.18)	0.01 (4.86)	0.09 (2.46)

### Family functioning in bereaved individuals

The mean score on family function (Family APGAR Index) was 6.5 (SD  =  2.7). 50% of the respondents (n  =  132) reported positive family function, 37.1% (n  =  98) moderate family dysfunction, and 12.9% (n  =  34) severe family dysfunction.

The mean scores on family cohesion and adaptability (FACES??) were 64.2 (SD  =  9.2) and 41.8 (SD  =  6.6), respectively. Compared to the Chinese norms on the FACES?? (cohesion: 63.9±8.0, adaptability: 50.9±6.2), the mean score on family adaptability in the study was significantly lower (t  =  –22.22, *p*  =  0.00), and the mean score on family cohesion was slightly higher but not statistically significant (t  =  0.56, *p*  =  0.58). Regarding the score on each item of the FACES??, the five items with the highest scores and the lowest scores are shown in Table 2. All the five items with the highest scores belonged to the family cohesion subscale. Based on linear scoring and interpretation of the FACES??, 15.9% (n  =  42) of the respondents belonged to extreme family type, 33.0% (n  =  87) mid-range family type, 46.2% (n  =  122) moderately balanced family type, and 4.9% (n  =  13) balanced family type.

**Table 2:** Five items with the highest and the lowest scores on the FACES?? (n  =  264).

Five items with highest scores	Mean (SD)	Five items with lowest scores	Mean (SD)
We approve of each other’s friends	3.95 (0.54)	Family members say what is on their minds	2.73 (0.87)
Family members are supportive of each other during difficult times	3.69 (0.74)	It is easier to discuss problems with people outside the family than with other family members	2.50 (0.97)
Family members feel very close to each other	3.67 (0.81)	In our family, it is easy for everyone to express his/her opinion	2.40 (0.91)
Family members go along with what the family decides to do	3.66 (0.60)	Our family members tries new ways of dealing with problems	2.36 (0.79)
Family members know each other’s friends	3.50 (1.00)	Family members feel closer to people outside the family than to other family members	2.29 (1.04)

### The relationships between demographic characteristics and disaster-related variables, and family functioning

The results of bivariate analyses found no statistically significant differences in family function (Family APGAR Index), cohesion, or adaptability (FACES??) between male and female respondents. No statistically significant differences were found in family function or family adaptability scores among different types of dead family members in the earthquake. Whether bereaved parents had another baby or were pregnant or not, and age groups were not significantly related to family adaptability. However, statistically significant differences were found in family function, cohesion, and adaptability depending on different educational levels, religious beliefs, marital status, financial loss during the earthquake, post-earthquake housing conditions, self-rated health status, and types of family structure (Table 1).

The results of multivariate regression analyses found that less financial loss in the earthquake was significantly related to positive family function (β =  –0.20, *p*  =  0.00). Relatively better health status after the earthquake was significantly related to positive family function, cohesion, and adaptability (β =  0.20, *p*  =  0.00; β =  0.22, *p*  =  0.00; β =  0.17, *p*  =  0.00). Scores on cohesion and adaptability among the respondents from nuclear families or extended families were significantly higher than those from single-parent families (β =  0.60, *p*  =  0.00; β =  0.56, *p*  =  0.00; β =  0.55, *p*  =  0.00; β =  0.48, *p*  =  0.00). Scores on family function and cohesion in bereaved parents who had another baby were significantly higher than those not pregnant (β =  0.13, *p*  =  0.04; β =  0.15, *p*  =  0.01) (Table 3).

**Table 3:** Multivariate regression analysis of predictors for family functioning (n  =  264).

Variables	Family function		Family cohesion		Family adaptability	
	β*	*p*	β*	*p*	β*	*p*
Demographic characteristics						
Gender (male/female)	–0.02	0.75	–0.03	0.57	0.02	0.79
Age (18–44/45–59/≥60years)	–0.04	0.68	–0.01	0.97	0.09	0.36
Educational level (illiteracy/primary school/junior high school/high school or above)	0.07	0.41	0.09	0.26	0.05	0.53
Religious beliefs (no/buddhism)	–0.10	0.10	–0.09	0.12	–0.15	0.08
Marital status (married/unmarried/divorced/windowed)	–0.10	0.18	–0.09	0.22	0.02	0.76
Disaster-related variables						
Financial loss during the earthquake (slight/moderate/severe)	–**0.20**	**0.00**	–0.09	0.15	–0.11	0.07
Types of dead family members in the earthquake						
Two or more than two types of family members	0.04	0.67	–0.05	0.53	–0.08	0.31
Spouse	0.14	0.08	–0.01	0.95	0.05	0.53
Children	0.06	0.58	–0.03	0.75	0.01	0.93
Reference group: other family members (parents, siblings, grandparents and grandchildren)						
Post-earthquake housing conditions (permanent/temporary)	–0.06	0.33	–0.05	0.39	–0.03	0.67
Post-earthquake self-rated health status (poor/moderate/good)	**0.20**	**0.00**	**0.22**	**0.00**	**0.17**	**0.01**
Post-earthquake types of family structure						
Nuclear family	0.22	0.08	**0.60**	**0.00**	**0.55**	**0.00**
Extended family	0.20	0.10	**0.56**	**0.00**	**0.48**	**0.00**
Reference group: single-parent family						
Post-earthquake fecundity status						
Pregnant	0.03	0.68	0.10	0.11	0.11	0.11
Already having another baby	**0.13**	**0.04**	**0.15**	**0.01**	0.12	0.06
Reference group: not pregnant						
Adjusted R^2^ (%)		16.5		26.4		14.3

* Standardized regression coefficients derived from multivariate linear regression

### The relationships between family functioning and loneliness

As shown in Table 4, positive family function, cohesion, and adaptability were significantly related to less emotional and social loneliness (r ranged from –0.31 to –0.53, *p* < 0.001), indicating that the better their perceived family functioning, the less emotional and social loneliness they experienced.

**Table 4:** Relationships between family functioning and loneliness (n  =  264).

Variables	Loneliness	Emotional loneliness	Social loneliness
Family function	–0.38***	–0.43***	–0.39***
Family cohesion	–0.35***	–0.53***	–0.40***
Family adaptability	–0.29***	–0.37***	–0.31***

*** *P* < 0.001

## Discussion

### Family functioning in bereaved individuals

Our study found that half of the bereaved individuals reported family dysfunction and 48.9% of the respondents reported a mid-range or extreme family type. An epidemiological study conducted by McDermott et al. (2010) found that three months after Cyclone Larry in Australia, the prevalence of family dysfunction in disaster-affected families was 28.6% [2], which is lower than our findings. The disparity may be attributed to the detrimental effects of disasters-induced bereavement on family functioning. Disaster deaths can disrupt family life. However, because no previous study has examined perceived family functioning in disaster bereaved individuals, our results cannot be compared meaningfully with findings from previous studies. Further studies are needed to compare perceived family functioning between bereaved and non-bereaved groups after disasters, and analyze whether bereavement caused by disasters is a risk factor for family health in disaster survivors.

We also found that all the five items with the highest scores in the FACES?? belonged to family cohesion dimension and the cohesion score was higher than the Chinese norms on the FACES??. This is consistent with Drabek’s view (1984) that emotional bonding, mutual support, as well as family ties tend to be reinforced after disasters [37]. Our findings are also in line with crisis theory developed by Andrew (1976), which suggests that although specific life events such as death or separation of family members can impede individuals’ satisfaction with basic needs, it can also improve family cohesiveness [38].

Another finding of this study was that the mean score on family adaptability of the sample was significantly lower than the Chinese norms on the FACES??. This concurs with the result of Dyregrov’s study (2001) that bereavement can influence family adaptation to traumatic events and grief over time [39]. Moreover, bereavement can interfere with individuals’ normal life [40]. The relationship between the dead and the bereaved does not disappear in a short time, and strong feelings for the lost person may last for many years, which may undermine ability of family members to transform existing family roles, role relationships, and power structure. How to accommodate to a new circumstance without the loved one or ones is a great challenge for bereaved individuals after disasters. Besides, all the respondents in our study experienced financial loss during the earthquake and over 40% of them lived in temporary post-disaster houses. This may contribute to the loss of safety and stability in the lives of disaster-affected families and result in difficulties for them in adapting to the tremendous family misfortune caused by a devastating natural disaster. In response, after the 2008 Wenchuan earthquake, central and local governments and nongovernmental organizations in China took various measures to help residents cope with disaster-induced health problems, such as sending rescuers and medical care teams to the earthquake-affected areas, transferring the wounded to other provinces for medical treatment, establishing mental health services stations and training residents for psychological relief. However, the majority of the mental health services were on the individual level but not on family health, suggesting the need to direct post-disaster psychotherapy, such as family therapy or family counseling, towards families and to nurture adaptation and development in families.

### Predictors for family functioning in bereaved individuals

Our study found that less financial loss during the earthquake was a significant predictor for positive family function. The result is similar to Norris and Uhl’s finding that greater financial loss predicted increased marital stress and reduced family support after hurricane Hugo [41]. Financial loss leads to economic pressure in the family, which could affect family functioning and individual adjustment. Although emergency assistance in food, drinking water, clothing, and medical care was provided by central and local governments immediately after the earthquake [42], long-term efforts to help survivors improve their economic situation are needed, especially for those with severe financial loss.

Perceived health refers to an individual's general perception of health, including biological, psychological, and social dimensions [43]. The present study found that better perceived health after the earthquake was a significant contributing factor to positive family function, cohesion, and adaptability. The result is consistent with the findings of Cano et al. (2003), who reported that poor self-rated health was a significant risk factor for family dysfunction in patients with chronic illnesses [44], and with the findings of McDermott and Cobham (2012) who reported that increased emotional distress in survivors was a significant risk factor for family dysfunction three months after Cyclone Larry in Australia [45]. Poor perceived health may increase family vulnerability with less employment opportunity, higher levels of family conflict, and increased resources consumption within the family. Improving the health status of family members might be an effective way to facilitate family functioning after disasters.

Our study also found that the respondents from nuclear families or extended families reported higher family cohesion and adaptability scores than those from single-parent families. This is consistent with Youngblut and Brooten’ study (2006)[12]. Nuclear family is characterized by independence and flexibility with more equal relationships shared by family members [46]. These characteristics may contribute to changes in relationship roles and family rules, and reduction in family conflict when traumatic events occur. In addition, in Chinese society, especially in the rural areas, extended family is the dominant family structure. In extended families, older parents usually cohabit with their children with strong emotional bonding. They are financially supported by their children and shoulder the responsibility for taking care of their grandchildren. This kind of family structure is conducive to developing close family ties and improving mutual negotiations and adaptability when an unpredictable crisis occurs. These findings suggest that more post-disaster efforts such as family therapy focusing on improving relationship patterns among family members should be provided for bereaved families, especially for single-parent families due to their lack of normal family structure and limited emotional support.

Moreover, our study found that those bereaved parents already having another baby after the earthquake reported significantly higher scores on family function and cohesion than those not pregnant. It was reported that about seventy thousand people died in the 2008 Wenchuan earthquake, and the vast majority of them were children and adolescents [26]. For the bereaved parents, the birth of another baby can bring hope and enhance emotional bonding among family members. However, as for the bereaved families who were still not pregnant eighteen months after the earthquake, their expectations of pregnancy were not satisfied. As a result, they might be filled with enormous stress, anxiety, and depression, which may impede emotional communication among family members and reduce family cohesion.

### The relationships between family functioning and loneliness

Although no previous study has explored the relationship between perceived family functioning and loneliness among disaster-affected individuals, the results of our study are in line with studies focusing on older people and patients with AIDS. Wu et al. (2010) found that positive family functioning and social support in the empty nest elderly were significant predictors for less loneliness [47]. Similar results were also found in the study of Sun et al. (2009) among AIDS patients [15]. These findings suggest that individuals with positive family functioning may obtain more emotional and spiritual support from family members and thereby reduce their loneliness. Bereavement can lead to family dysfunction. Impaired family functioning can in turn result in loneliness. Further studies are needed to explore the moderating effect of family functioning on the relationship between disaster-related bereavement and loneliness.

Several limitations are identified in the study. First, participants of this study were drawn from the hardest-hit areas and a convenience sampling method was used, which might have undermined the representativeness of the selected sample. Second, the study adopted a cross-sectional design, which excluded the possibility of identifying possible developmental variations in perceived family functioning. A longitudinal study is warranted. Third, perceived family functioning of the participants before the earthquake was not assessed. This made it impossible to compare the reported prevalence of family dysfunction with that before the earthquake. Therefore, the reported prevalence of family dysfunction should be interpreted with caution. Finally, only a small number of predictors for family functioning in bereaved individuals were examined in our study. Future research needs to investigate other possible predictors, such as coping styles and social support.

Despite these limitations, to our knowledge, this is the first study exploring family functioning and its predictors as well as the relationship between family functioning and loneliness in disaster bereaved individuals. Results of this study provide new insight into the psychosocial aftermath of catastrophic natural disasters. Specifically, the study revealed a high prevalence of family dysfunction in disaster bereaved individuals eighteen months after the Wenchuan earthquake and identified four significant predictors for perceived family functioning. These findings can provide useful implications for post-disaster rebuilding and relief work, particularly for healthcare providers to identify high-risk bereaved populations and to develop strategies to help them. Such strategies may include helping disaster survivors with financial difficulties to improve their economic situation, providing long-term health care to those with poor health status, providing professional reproductive assistance to bereaved parents, and initiating family psychotherapy such as family counseling for single-parent families to improve family functioning and reduce loneliness for bereaved individuals.
